# Supplementary material for: Base-pair resolution analysis of the effect of supercoiling on DNA flexibility and major groove recognition by triplex-forming oligonucleotides
Source: Nat Commun. 2021 Feb 16;12:1053. doi: 10.1038/s41467-021-21243-y (PMC7887228; doi:10.1038/s41467-021-21243-y)
Supplement: Supplementary file 3 — Description of Additional Supplementary Files [file 41467_2021_21243_MOESM3_ESM.docx]

**Description of Additional Supplementary Files**

File Name: *Supplementary Video 1*

Description: Rotational movie of the averaged structure for topoisomer ΔLk = 0 obtained from simulations (Fig. 2a) showing the degree planarity on the conformation, where the average and maximum deviation from the best fitting plane along the minicircle are 3% and 7%, respectively (see Figure S1).

File Name: *Supplementary Video 2*

Description: Rotational movie of the averaged structure for topoisomer ΔLk = -1 obtained from simulations (Fig. 2a) showing the degree of planarity on the conformation, where the average and maximum deviation from the best fitting plane along the minicircle are 12% and 25%, respectively (Figure S1).

File Name: *Supplementary Video 3*

Description: Rotational movie of the averaged structure for topoisomer ΔLk = -2 obtained from simulations (Fig. 2a) showing the degree of planarity on the conformation, where the average and maximum deviation from the best fitting plane along the minicircle are 5% and 12%, respectively (Figure S1).

File Name: *Supplementary Video 4*

Description: Rotational movie of the averaged structure for topoisomer ΔLk = -3 obtained from simulations (Fig. 2a) showing the degree of planarity on the conformation, where the average and maximum deviation from the best fitting plane along the minicircle are 4% and 11%, respectively (Figure S1).

File Name: *Supplementary Video 5*

Description: Rotational movie of the averaged structure for topoisomer ΔLk = -6 obtained from simulations (Fig. 2a) showing the degree of planarity on the conformation, where the average and maximum deviation from the best fitting plane along the minicircle are 4% and 10%, respectively (Figure S1).

File Name: *Supplementary Video 6*

Description: *A trajectory fragment 400 picosecond (ps) long extracted from the implicitly-solvated MD simulation of the 339 bp ΔLk -1 topoisomer mirroring the dynamics observed by AFM in Figure 1f (top sequence of structures).* A series of four snapshots extracted from this trajectory equally-spaced at 100 ps are sketched on the top row of Figure 1g. Note that timescales are not physically meaningful because water viscosity is disregarded in the used model of implicit representation of the solvent.

File Name: *Supplementary Video 7*

Description: *A second trajectory fragment 300 ps long extracted from the implicitly-solvated MD simulation of the 339 bp ΔLk -1 topoisomer mirroring the dynamics observed by AFM in Figure 1f (bottom sequence of structures).* A series of four snapshots extracted from this trajectory equally-spaced 75 ps are sketched on the bottom row of Figure 1g. As before, timescales are not physically meaningful.
