# Supplementary material for: Base-pair resolution analysis of the effect of supercoiling on DNA flexibility and major groove recognition by triplex-forming oligonucleotides
Source: Nat Commun. 2021 Feb 16;12:1053. doi: 10.1038/s41467-021-21243-y (PMC7887228; doi:10.1038/s41467-021-21243-y)
Supplement: Supplementary file 11 — Reporting Summary [file 41467_2021_21243_MOESM11_ESM.pdf]

nature research

Corresponding author(s): Alice Pyne

Last updated by author(s): Oct 30, 2020

Reporting Summary

Nature Research wishes to improve the reproducibility of the work that we publish. This form provides structure for consistency and transparency in reporting. For further information on Nature Research policies, see our Editorial Policies and the Editorial Policy Checklist.

Statistics

For all statistical analyses, confirm that the following items are present in the figure legend, table legend, main text, or Methods section.

n/a	Confirmed	
☐	☒	The exact sample size (n) for each experimental group/condition, given as a discrete number and unit of measurement
☐	☒	A statement on whether measurements were taken from distinct samples or whether the same sample was measured repeatedly
☒	☐	The statistical test(s) used AND whether they are one- or two-sided<br>*Only common tests should be described solely by name; describe more complex techniques in the Methods section.*
☒	☐	A description of all covariates tested
☒	☐	A description of any assumptions or corrections, such as tests of normality and adjustment for multiple comparisons
☐	☒	A full description of the statistical parameters including central tendency (e.g. means) or other basic estimates (e.g. regression coefficient) AND variation (e.g. standard deviation) or associated estimates of uncertainty (e.g. confidence intervals)
☒	☐	For null hypothesis testing, the test statistic (e.g. F, t, r) with confidence intervals, effect sizes, degrees of freedom and P value noted<br>*Give P values as exact values whenever suitable.*
☒	☐	For Bayesian analysis, information on the choice of priors and Markov chain Monte Carlo settings
☒	☐	For hierarchical and complex designs, identification of the appropriate level for tests and full reporting of outcomes
☒	☐	Estimates of effect sizes (e.g. Cohen's d, Pearson's r), indicating how they were calculated

Our web collection on statistics for biologists contains articles on many of the points above.

Software and code

Policy information about availability of computer code

Data collection	Gromacs4.5 was used for running all simulations except the ones with bsc1 force-field, which were run in Amber16<br>AFM data was collected on Bruker Multimode 8 and FastScan Bio systems, using their proprietary software Nanoscope (v9.2 &v 9.5).
Data analysis	Amber12 software packaged was used for preparation and analysis of simulations<br>WrLINE1.0 and SerraLINE1.0 were used for writhe and bending calculations and are available at https://github.com/agnesnoy<br>Chimera1.14 and VMD1.9.3 were the visualizing software used for creating images of structures from from molecular dynamics simulations<br>AFM data was analysed using Gwyddion software (v2.45) and python (v2.7) scripting available at https://github.com/AFM-SPM/TopoStats<br>Gel electrophoresis images were analysed using ImageQuant (v7.0)

For manuscripts utilizing custom algorithms or software that are central to the research but not yet described in published literature, software must be made available to editors and reviewers. We strongly encourage code deposition in a community repository (e.g. GitHub). See the Nature Research guidelines for submitting code & software for further information.

Data

Policy information about availability of data

All manuscripts must include a data availability statement. This statement should provide the following information, where applicable:

- Accession codes, unique identifiers, or web links for publicly available datasets
- A list of figures that have associated raw data
- A description of any restrictions on data availability

All data generated and/or analysed during the current study are available in the figshare repository https://doi.org/10.6084/m9.figshare.13116890. This includes the raw data for each figure in the manuscript and SI.

Field-specific reporting

Please select the one below that is the best fit for your research. If you are not sure, read the appropriate sections before making your selection.

- [x] Life sciences
- [ ] Behavioural & social sciences
- [ ] Ecological, evolutionary & environmental sciences

For a reference copy of the document with all sections, see nature.com/documents/nr-reporting-summary-flat.pdf

Life sciences study design

All studies must disclose on these points even when the disclosure is negative.

Sample size	For AFM: Sample sizes were based on reproducibility of the result and on previous experience. Each experiment was repeated multiple times, and showed the same trend when analysed using automated code. For high resolution measurements, less repeats were obtained due to the difficulty of these measurements, however, multiple molecules from at least two samples were analysed. The sample size used for AFM was larger than or equivalent to others in the field (Nievergelt, 2018), (Parsons, 2019).<br>For MD simulations: 3000 frames were taken from last 30 ns of each simulation every 10 ps for subsequent analysis. Tests were done using 30000 values or the last 20 ns with no significant difference. The only exception is Figure 2d where B-DNA bends stronger than 30 degrees (in total 23) and all kinks (10) were used. Note each bend value was obtained following the previous rules. The sample sizes used for the MD simulations are equivalent to those in the literature (Irobalieva, 2015)
Data exclusions	For AFM analysis of the effect of supercoiling on the overall structure of DNA minicircles, only data taken using the same immobilisation and imaging method was used. This was to ensure results were comparable across multiple datasets. Images were only excluded for AFM if the data quality was too poor to allow the data to be automatically processed, and therefore allowed for consistent exclusion reducing bias.
Replication	Two MD replicas were run for the -2 and the -3 topoisomers successfully being presented in the current study, MD replicas were successful. AFM replicas were performed successfully with the same results over a period of four years by two separate co-authors.
Randomization	Randomization was not carried out due to the design of the study. In both AFM and MD experiments were designed to probe a specific outcome, and therefore could not be randomized. Images were analysed using automated code to reduce bias.
Blinding	Blinding was not employed due to the unambiguous nature of the measurements, these measurements were assessed using automated code, and therefore blinding would not have reduced bias. .

Reporting for specific materials, systems and methods

We require information from authors about some types of materials, experimental systems and methods used in many studies. Here, indicate whether each material, system or method listed is relevant to your study. If you are not sure if a list item applies to your research, read the appropriate section before selecting a response.

Materials & experimental systems

n/a	Involved in the study	
☒	☐	Antibodies
☒	☐	Eukaryotic cell lines
☒	☐	Palaeontology and archaeology
☒	☐	Animals and other organisms
☒	☐	Human research participants
☒	☐	Clinical data
☒	☐	Dual use research of concern

Methods

n/a	Involved in the study	
☒	☐	ChIP-seq
☒	☐	Flow cytometry
☒	☐	MRI-based neuroimaging
